# Anthropogenic noise increases fish mortality by predation

**DOI:** 10.1038/ncomms10544

**Published:** 2016-02-05

**Authors:** Stephen D. Simpson, Andrew N. Radford, Sophie L. Nedelec, Maud C. O. Ferrari, Douglas P. Chivers, Mark I. McCormick, Mark G. Meekan

**Affiliations:** 1 Biosciences, College of Life and Environmental Sciences, University of Exeter, Geoffrey Pope, Stocker Road, Exeter EX4 4QD, UK; 2 School of Biological Sciences & Cabot Institute, University of Bristol, 24 Tyndall Avenue, Bristol BS8 1TQ, UK; 3 Department of Biomedical Science, Western College of Veterinary Medicine, University of Saskatchewan, Saskatchewan, Canada S7N 5B4; 4 Department of Biology, University of Saskatchewan, Saskatchewan, Canada S7N 5E2; 5 Australian Research Council Centre of Excellence for Coral Reef Studies and School of Marine and Tropical Biology, James Cook University, Townsville, Queensland 4811, Australia; 6 Australian Institute of Marine Science, Perth, Western Australia 6009, Australia

## Abstract

Noise-generating human activities affect hearing, communication and movement in terrestrial and aquatic animals, but direct evidence for impacts on survival is rare. We examined effects of motorboat noise on post-settlement survival and physiology of a prey fish species and its performance when exposed to predators. Both playback of motorboat noise and direct disturbance by motorboats elevated metabolic rate in Ambon damselfish (*Pomacentrus amboinensis*), which when stressed by motorboat noise responded less often and less rapidly to simulated predatory strikes. Prey were captured more readily by their natural predator (dusky dottyback, *Pseudochromis fuscus*) during exposure to motorboat noise compared with ambient conditions, and more than twice as many prey were consumed by the predator in field experiments when motorboats were passing. Our study suggests that a common source of noise in the marine environment has the potential to impact fish demography, highlighting the need to include anthropogenic noise in management plans.

## Abstract (web summary)

Little evidence exists on whether human-generated noise directly affects survival of wildlife. Here, Simpson *et al*. show that damselfish exposed to motorboat noise have elevated metabolic rates, reduced responses to predatory attacks, and suffer increased predation compared to fish in ambient conditions.

## Graphical abstract

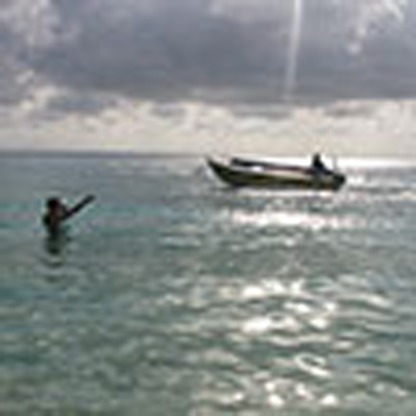

Since the Industrial Revolution, anthropogenic (man-made) noise has changed the soundscape of many terrestrial and aquatic ecosystems^1^,^2^,^3^. International legislation, such as the US National Environment Policy Act and the European Commission Marine Strategy Framework Directive, recognizes the need to assess and manage the biological impacts of noise-generating human activities. However, while recent studies have demonstrated that anthropogenic noise can detrimentally affect animal hearing thresholds, communication, movement patterns and foraging^3^,^4^,^5^, it is often difficult to translate these effects into meaningful predictions about individual fitness and population-level consequences^6^,^7^. This is because animals may be able to move away from noise sources, acoustic disturbance may be sporadic, and compensation by organisms may prevent long-term impacts^3^,^6^,^8^. Recent correlative evidence suggests that naval sonar may cause mortality in beaked whales^9^, but it is impossible to test this directly. Thus, there is a clear need for experimental studies on tractable organisms that investigate directly whether common sources of anthropogenic noise reduce survival.

In marine environments, noise pollution is derived from a variety of sources including pile-driving, seismic surveys, shipping and motorboat traffic^3^,^4^. Much of this noise occurs in coastal regions, which are experiencing unprecedented human population growth^10^ and thus significant rises in transportation, fishing and recreation activities that involve boating^11^. For example, there were >12.5 million registered motorboats in the USA in 2013 (ref. 12) and there are expected to be 0.5 million recreational motorboats using the Great Barrier Reef by 2040 (ref. 13). Motorboats are therefore a prevalent and increasing source of anthropogenic noise, with emerging evidence that this noise could affect communication, orientation and territorial behaviour in fish^14^. Unlike industrial sources of noise such as pile-driving and commercial shipping, it is relatively straightforward to design studies that use motorboats in controlled experiments to test impacts of noise on marine organisms.

Here, we examine the effect of motorboat noise on predator–prey dynamics and survivorship using a model coral reef system that lends itself to manipulation, observation and replication: the Ambon damselfish *Pomacentrus amboinensis* and its predator, the dusky dottyback *Pseudochromis fuscus*. Damselfishes share life-history traits with the majority of benthic and coastal fishes and invertebrates, typified by demersal, site-attached adults that produce pelagic larvae that develop in open water before settling to suitable habitat where they will live as juveniles and adults^15^. On settlement to reef habitat, young naïve fish encounter a suite of novel predators and suffer high rates of mortality that make the first few days post-settlement a critical population bottleneck^16^. We tested the impact of motorboat noise on the post-settlement survival, physiology and performance of *P. amboinensis* when exposed to the predator *P. fuscus*, thus providing a direct assessment of the fitness consequences of anthropogenic noise. We found that both motorboat noise and direct disturbance by motorboats elevated stress and reduced anti-predator responses, more than doubling mortality by predation.

## Results

### Survival on patch reefs

**Figure 1:**
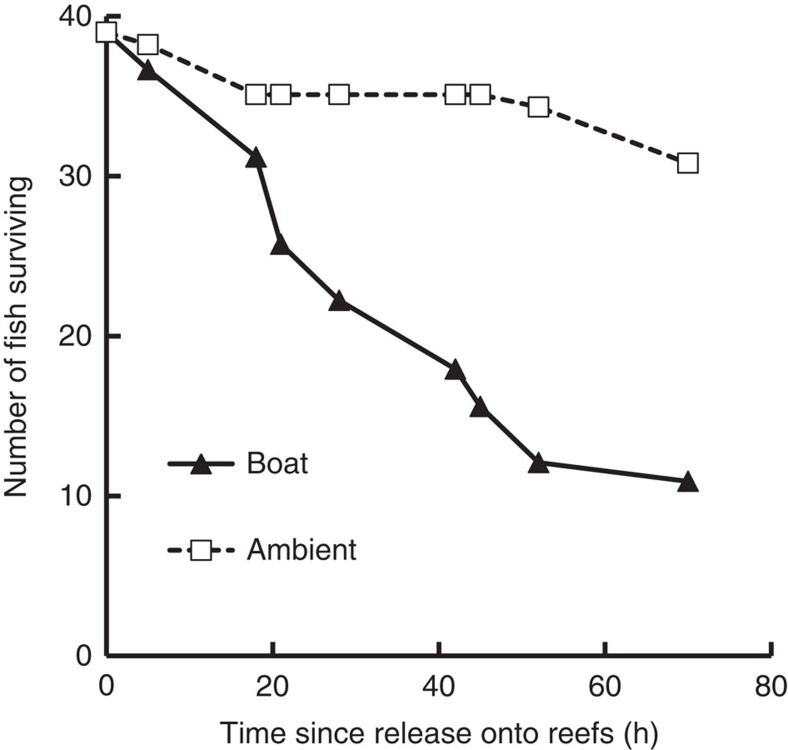
Survival of *P. amboinensis* on reefs with and without playback of boat noise. Field-based survival of *P. amboinensis* during 72 h following release onto experimental patch reefs with playback of ambient or boat-noise recordings using underwater speakers.

*P. amboinensis* suffers natural mortality rates of ∼50% in the first 5 days after settlement to reef habitat^17^. We tested whether motorboat noise affected the likelihood of mortality at this time by placing settlement-stage individuals on small isolated experimental patch reefs on sandflats (as per ref. 18), with underwater sound systems broadcasting either recordings of ambient habitat noise or ambient noise with the addition of noise from motorboats passing nearby (10–200 m away). Addition of boat noise in the playback recordings had a significant negative effect on survival of *P. amboinensis* (Cox's *F*=4.30, *n*_amb_, *n*_boat_=39 fish on individual reefs, *P*<0.001): 79% of recruits survived the 72 h observation period in the control treatment, but only 27% survived in the boat-noise treatment (Fig. 1). It is possible that some of this additional mortality was directly caused by noise-induced physiological changes^19^. However, given that mortality at this life-history stage is driven predominantly by predation^18^, we used a series of further experiments to examine the factors driving increased predation and to assess the consequences of boat noise on predator–prey interactions and prey fitness.

### Metabolic rate

**Figure 2:**
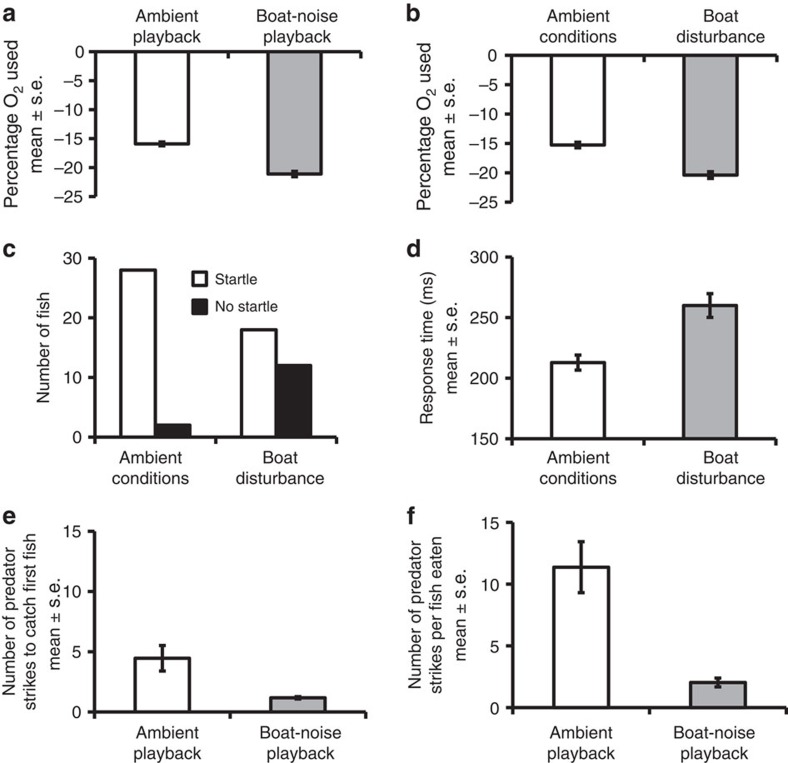
Impacts of boat noise on active metabolic rate and anti-predator response of *P. amboinensis*, and predatory success on *P. amboinensis* by its natural predator *P. fuscus*. (**a**) Mean±s.e. oxygen depletion for *P. amboinensis* exposed to playback of ambient or boat-noise recordings in tanks (*n*=29 for each treatment). (**b**) Mean±s.e. oxygen depletion for *P. amboinensis* exposed to ambient conditions or boats motoring nearby (*n*=19 for each treatment). (**c**) Number of *P. amboinensis* exhibiting a startle response to a looming stimulus with or without boats motoring nearby (*n*=30 for each treatment). (**d**) Mean±s.e. time taken to startle to a looming stimulus by those individuals in **c** that exhibited a startle response (ambient conditions: *n*=28; boat motoring nearby: *n*=18). (**e**) Mean±s.e. number of strikes made by *P. fuscus* to catch the first *P. amboinensis* while exposed to playback of ambient or boat-noise recordings in tanks (*n*=18 for each treatment). (**f**) Mean±s.e. number of strikes per prey item in the same experiment.

Recent laboratory work on fish has established the possibility that anti-predator behaviour in European eels (*Anguilla anguilla*) could be compromised by a noise-induced elevation in stress, as indicated by an increase in active metabolic rate of individuals in sealed tubes exposed to different playbacks of noise^20^. We used the same approach and found that settlement-stage *P. amboinensis* used 20% more oxygen over 30 min when experiencing playback of motorboat noise compared with playback of ambient noise (independent samples *t*-test: *t*=9.04, df=57, *n*_amb_=30, *n*_boat_=29, *P*<0.001; Fig. 2a). Since playback of field recordings in tanks do not fully replicate the acoustic conditions in open water with real noise sources, we repeated this study *in situ* with motorboats. We found a similar result using the same experimental design: settlement-stage *P. amboinensis* used significantly more (33%) oxygen when exposed to motorboats passing compared with ambient conditions (*t*=6.29, *n*_amb_, *n*_boat_=18, *P*<0.001; Fig. 2b).

### Anti-predator behaviour

Noise-induced stress could reduce the likelihood that prey detect the approach of predators, and thus do not react with an appropriately rapid startle response^21^,^22^. We designed a looming-stimulus experiment (based on ref. 23) for use with motorboats in open water to test the effect of boat noise on the anti-predator behaviour of settlement-stage *P. amboinensis*. We found that when motorboats were passing, *P. amboinensis* were six times less likely to startle to a simulated predator attack compared with fish tested in ambient conditions (Fisher's exact test: *n*_amb_, *n*_boat_=30, *P*=0.005; Fig. 2c). Of those fish that startled, the response time after the release of the stimulus was 22% slower (independent samples *t*-test: *t*=4.28, *n*_amb_=28, *n*_boat_=18, *P*<0.001; Fig. 2d) with motorboats passing compared with ambient conditions. Consequently, the ‘predator' was 31% closer to fish that startled during exposure to boat noise (mean±s.e.: 4.17±0.26 cm) than in ambient conditions (2.56±0.28 cm; *t*=4.11, *P*<0.001).

### Strike–success rate of predators

The extent to which such a reduction in anti-predator responses could influence the survival of prey also depends on the impact of boat noise on the performance of the predator. Therefore, we observed interactions between *P. fuscus* and *P. amboinensis*, an established predator–prey model system^24^, in predation trials conducted with and without motorboat noise. To allow detailed observation, we initially conducted the experiment in tanks (as per ref. 24), using playback of field recordings. Predation by *P. fuscus* was more successful in trials with boat-noise playback than in trials with ambient-noise playback, with the predators needing 74% fewer strikes to capture their first prey (Mann–Whitney *U*-test: *U*=34.5, *n*_amb_, *n*_boat_=18, *P*<0.001; Fig. 2e) and 82% fewer strikes per prey capture overall (Mann–Whitney *U*-test: *U*=27.5, *n*_amb_, *n*_boat_=18, *P*<0.001; Fig. 2f).

### Mortality due to predation

**Figure 3:**
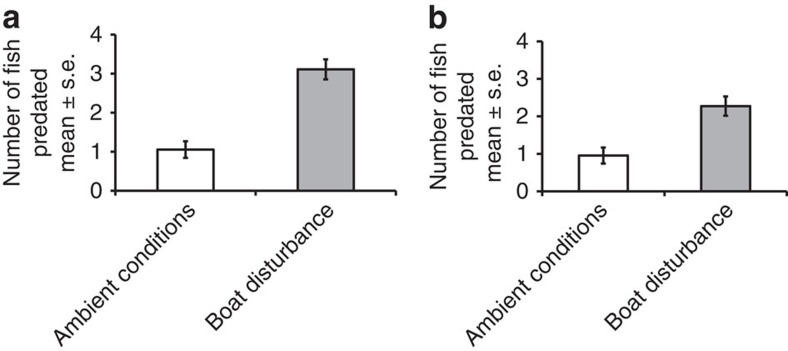
Direct impact of boat noise on predation of *P. amboinensis* by its natural predator *P. fuscus*. (**a**) Mean±s.e. number of *P. amboinensis* eaten of 10 individuals during 15 min trials in controlled tank conditions, with and without playback of ambient or boat-noise recordings. (**b**) Mean±s.e. number of *P. amboinensis* eaten from five individuals during 15 min trials in open-water conditions, with and without boats motoring nearby.

To examine the consequences of these noise-related effects for the fitness of *P. amboinensis*, we investigated survival likelihood in 15 min predator–prey trials. In the tank-based experiment, there was a significant effect of boat-noise playback (MannWhitney *U*-test: *U*=52, *n*_amb_, *n*_boat_=18, *P*<0.001), with 2.9 times as many *P. amboinensis* consumed compared with when there was playback of ambient noise (Fig. 3a). Importantly, in a similar experiment in open water, 2.4 times as many *P. amboinensis* were consumed by *P. fuscus* when motorboats were passing compared with ambient conditions (*U*=99, *n*_amb_, *n*_boat_=22, *P*<0.001; Fig. 3b).

## Discussion

Our study demonstrates a direct impact of anthropogenic noise on predator–prey dynamics and quantifies, for the first time, the negative consequences for prey survival. Combining laboratory and field experiments, utilizing playbacks and real noise sources, we provide strong evidence that motorboat noise can have detrimental effects on anti-predator behaviour, potentially as a result of increased stress. In our model system, boat noise favoured the predator, with the prey suffering reduced fitness; the winners and losers in other predator–prey interactions will depend on the relative hearing sensitivities and noise tolerances of the species involved, as well as the particular noise source. For instance, previous tank-based playback studies have shown that additional noise can reduce the foraging success of fish and crabs^25^,^26^, although whether this translates into consequences for fitness is unknown. It remains to be determined whether repeated exposure to boat noise would result in increased tolerance by *P. amboinensis*, and thus a lessened impact over time^8^. However, tolerance can only develop, and compensation is only possible, in animals that survive predatory attacks.

Elevated stress in response to noise was indicated by the increased active metabolic rate of *P. amboinensis*^27^; previous work has also suggested that noise can cause stress in fish^20^,^28^. A general allostatic stress response could result in decreased locomotor activity^29^ or affect attention^22^. Both are possible explanations for the reduced likelihood of startle responses by prey to predatory attacks, also seen in laboratory work on eels and crabs utilizing playback and simulated predator assays^20^,^26^. Reduced performance of prey in response to a predator could also arise from noise-induced distraction^30^ or from the masking of acoustic cues indicating the approach of a predator^31^.

In this study, we focussed on the critical life-history period immediately following settlement of larval reef fish to benthic habitat. Our experiments suggest that motorboat noise could increase mortality at this transitional stage; future experiments are needed to test the spatial scale of impact for a range of species at key life-history stages. In those coral reef environments where motorboat noise is a frequent event, for example the Great Barrier Reef, this level of disturbance could affect the demography of impacted populations. A range of stressors increasingly threatens coral reefs (refs 32, 33), yet reefs generate important revenue for many countries through tourism, and provide food and livelihoods through fisheries to 500 million people^33^. If sufficient resilence is to be retained for reef ecosystems to survive predicted global climate change, managing current and local environmental stressors has been proposed as an essential goal. Our work highlights the need for anthropogenic noise to be included in environmental management plans and, in general, the importance of assessing the direct fitness consequences of anthropogenic noise.

## Methods

### Permits and ethical approval

This study was conducted during October–November in 2012 and 2014 at Lizard Island Research Station (14° 40′ S, 145° 28′ E), Great Barrier Reef, Australia, with permission and ethical approval from: Lizard Island Research Station, Great Barrier Reef Marine Park Authority, James Cook University (A2081), Australian Institute of Marine Science and University of Exeter (2013/247).

### Acoustic stimuli and playback conditions

Daytime ambient recordings were made in the bay in front of Lizard Island Research Station (where all field trials were conducted) at five different inshore sandy-bottom locations, at 3–5 m depth and always >100 m from reefs. At each location, a recording was also made with one of five of the research station boats (5-m-long aluminium hulls with 30 hp Suzuki outboard motors) motoring at various speeds 10–200 m from the hydrophone and accelerometer, replicating the kinds of boat operations common in coral reef environments. Much of the Great Barrier Reef lagoon is <40 m deep and fishermen, divers and tourists typically operate motorboats in shallow water (<10 m deep) over and through reefs. At popular sites on the Great Barrier Reef, many boats may pass over a reef each hour. Recordings were taken from a kayak moored using an anchor without chain to avoid unwanted noise (for example, waves on the hull of a boat), and were made 1 m above the seabed for 5 min. Acoustic pressure was measured using a calibrated omnidirectional hydrophone (HiTech HTI-96-MIN with inbuilt preamplifier, manufacturer-calibrated sensitivity-164.3 dB re 1 V/μPa; frequency range 0.02–30 kHz; calibrated by manufacturers; High Tech Inc., Gulfport MS) and a digital recorder (PCM-M10, 48 kHz sampling rate, Sony Corporation, Tokyo, Japan). Particle acceleration was measured using a calibrated triaxial accelerometer (M20L; sensitivity following a curve over the frequency range 0–3 kHz; calibrated by manufacturers; Geospectrum Technologies, Dartmouth, Canada) and a digital 4-track recorder (Boss BR-800, 44.1 kHz sampling rate, Roland Corporation, Los Angeles, CA). Recording levels used with each set-up were calibrated using pure sine wave signals from a function generator with a measured voltage recorded in line on an oscilloscope.

For the playback experiments, a unique compilation of three of the five ambient recordings (made using Audacity v2.0.2, http://audacity.sourceforge.net) was used for each control track, and compilations of three of the five boat-noise recordings made at the same five locations were used for the boat-noise tracks. The sound systems used for playback of ambient and boat-noise recordings consisted of a battery (12v 7.2 Ah sealed lead-acid), WAV/MP3 player (GoGEAR Vibe, frequency response 0.04–20 kHz; Philips, The Netherlands), amplifier (M033N, 18 W, frequency response 0.04–20 kHz; Kemo Electronic GmbH, Germany) and speaker (University Sound UW-30; maximal output 156 dB re 1 μPa at 1 m, frequency response 0.1–10 kHz; Lubell Labs, Columbus, OH).

**Figure 4:**
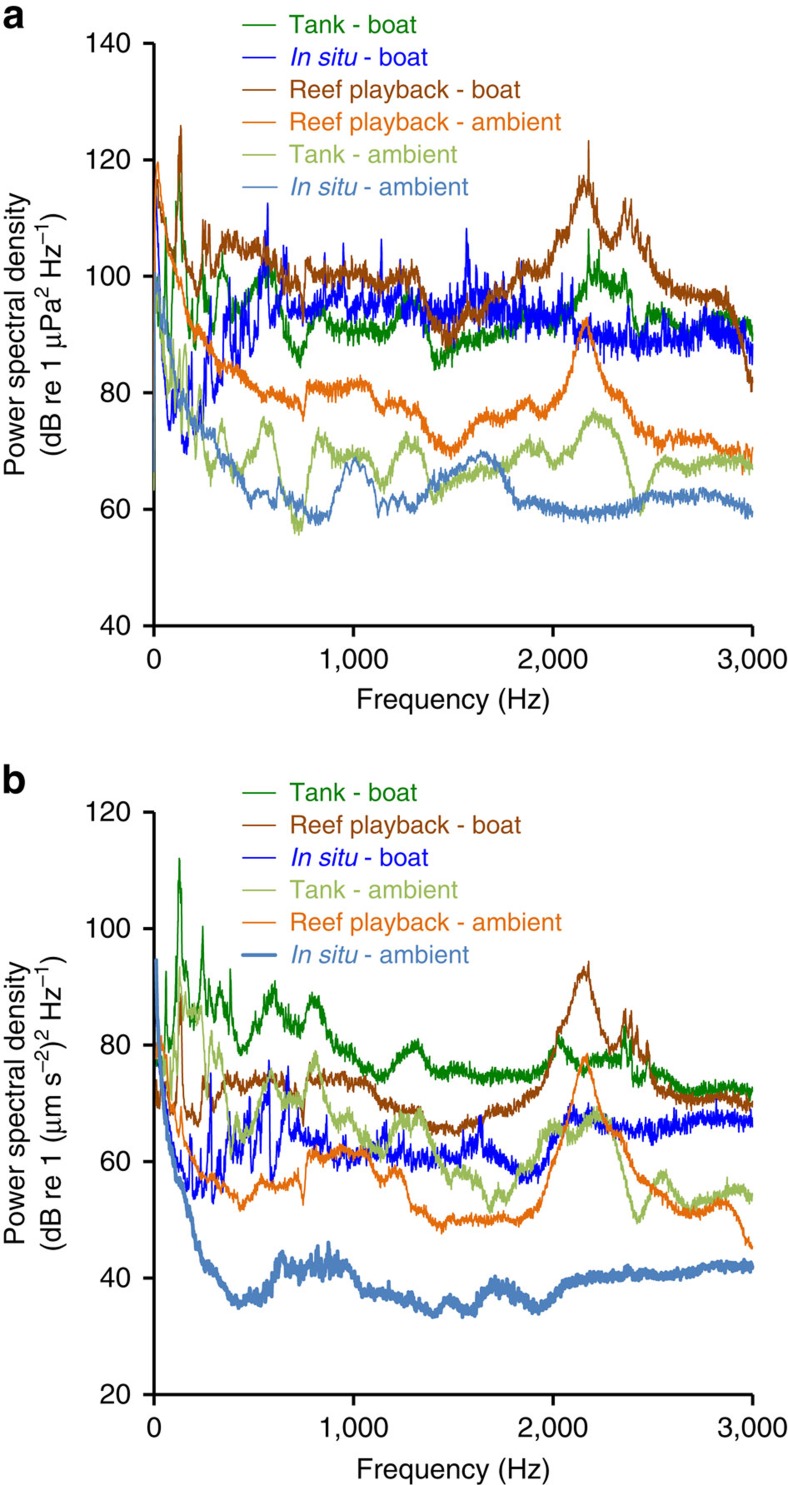
Analysis of acoustic stimuli and playback conditions. Spectral content shown in both (**a**) acoustic pressure and (**b**) particle acceleration domains for original field recordings of boat noise and ambient conditions, playback of ambient and boat-noise recordings in the field, and playback of ambient and boat-noise recordings in experimental tanks. Mean power spectral density of 1 min of each sound condition is shown. In both the pressure and particle acceleration domains, there is a clear difference between boat and ambient conditions, whether using playback or real noise, and in tanks or *in situ*. However, received levels of particle acceleration at the fish were higher in playback treatments than with motorboats. Sounds were analysed in MATLAB v2010a, fft length=sampling frequency (48 kHz for sound pressure and 44.1 kHz for particle acceleration, both result in 1 Hz bands).

Ambient and boat-noise recordings, and recordings of their playback in open water and in experimental tanks (descriptions below), were analysed using MATLAB v2010a: Fast-Fourier Transformation was used to calculate power spectral density for comparison of sound levels for each treatment across the frequency range 0–3000 Hz (Fig. 4). Playback using speakers in both natural settings and in tanks alters the characteristics of the original recordings. However, analysis of spectral content and sound levels showed that characteristics of the original recordings were at least partially retained in playback and that these characteristics differed between playback of ambient and motorboat noise in both the field and in tanks. As boat-noise playbacks in the tank were too loud to characterize fully using the accelerometer due to instrument sensitivity (‘clipping' was observed), we could not describe the full extent of particle acceleration levels, although it is clear that they were louder than the ambient playback.

### Impact of boat noise on survival on patch reefs

Following a ∼3-week pelagic larval stage, young *P. amboinensis* settle onto patch reefs and continuous reefs in shallow waters 1–15 m deep. In this novel habitat, juveniles are exposed to a diverse range of predators that use both ambush (lizardfish *Synodus dermatogenys* and small cods *Cephalopholis microprion*) and pursuit (dottybacks *P. fuscus* and wrasse *Thalassoma lunare*) tactics. These species are regularly observed capturing juvenile reef fish that venture away from shelter^34^, and all species were seen around the experimental reefs during this experiment. We tested the effect of boat-noise playback on the mortality rate of settlement-stage *P. amboinensis* released onto experimental patch reefs.

Our experimental reefs comprised pieces of healthy and dead bushy hard coral *Pocillopora damicornis* (∼18 × 15 × 18 cm) placed on sandflats. Ten such reefs were spaced 3 m apart in a circle at four different shallow-water (3–5 m) sites that were separated by >400 m on the backreef of the fringing reef. At each site, a sound system (details above) was moored so that the speaker was suspended in the centre of the reefs. Patches were cleared of any fishes or large invertebrates using hand nets before release of experimental fish. Sound systems played either tracks of ambient noise or alternated between tracks of 5 min ambient and 5 min boat noise (details above).

Settlement-stage fishes were collected overnight using light traps^35^ moored ∼500 m offshore in open water (∼10–20 m depth) around Lizard Island, transported in 60 l tubs back to the research station where they were sorted to species and *P. amboinensis* were transferred to 30 l aquaria supplied with a continuous flow of aerated seawater. Fish were held for 4 days (27–30 °C, 14:10 h natural light:dark cycle) and fed twice daily *ad libitum* with newly hatched *Artemia* sp. On the day of the release, *P. amboinensis* were placed into individually labelled 1 l plastic bags of seawater and kept in a water bath of flowing seawater until deployment in the field. To allow the identification of experimental fish from any other fish that might naturally recruit to our experimental reefs, fish were tagged subcutaneously with a red fluorescent elastomer tattoo using a 27-gauge hypodermic needle (as per ref. 33). This left a visible 1.5–2 mm-long stripe of colour on the flank of the fish. Tagging with a single elastomer tattoo has been found to have no influence on the mortality or growth of this species^36^, but has demonstrated that loss of individuals from reefs is not due to post-settlement migrations^37^. Fish were transported in bags to the field site in a shaded 60 l water bath to maintain a stable temperature and offer conditions of diffused light to minimize stress of transfer.

A single *P. amboinensis* was selected at random and placed on each of the 10 reefs at the four sites, and mortality was then monitored twice daily for 3 days. The experiment was repeated to control for any site effects, with the sound treatments (that is, boat-noise playback with ambient reef sound, or playback of ambient reef sound) at each site reversed for the second block. Survival (up to 72 h) of *P. amboinensis* in the two acoustic treatments was compared using multiple-sample Survival Analysis, which uses a Cox's proportional hazard model (Statistica 9.0). Survival curves for fish within each treatment were calculated and plotted using the Kaplan–Meier product-limit method, which is a non-parametric estimator of survival that incorporates incomplete (censored) observations, such as those cases where fish had not died by the end of the census period. The difference in survival of fish between the boat-noise and ambient-noise treatments was compared using the Cox–Mantel test with a Cox's *F* statistic.

### Impact of boat noise on metabolic rate

The methods for assessing metabolic rate from oxygen depletion followed those of Simpson *et al.*^20^ Two experiments were conducted: the first, in tanks, examined the impact of playback of boat noise; the second, in the field, examined the impact of motorboats. For both experiments, *P. amboinensis* were collected in light traps (as above), and were starved for 20 h before the experiment to avoid any inter-individual effects of metabolic demands due to digestion^38^. At the start of the experiment, individual fish were randomly allocated to each treatment, to avoid any biases arising from preferential capture. Each fish was transferred by a scoop and sealed in a weighted 120 ml opaque plastic tube (12 cm long, estimated to be 90–95% acoustically transparent based on typical acoustic impedance of polystyrene versus water). The tube was filled with fully aerated seawater (89–91% O_2_ saturation, 28–29 °C), with the top sealed underwater to avoid air bubbles. Dissolved O_2_ content of the water was measured (Dissolved Oxygen and Temperature Meter HI 9164, Hanna Instruments Inc., Woonsocket, RI) before and after 30 min of exposure to sound. After each trial, fish were weighed and measured to test for bias in the size of fish allocated to different treatments.

In the first experiment, four exposure tanks were used; these were placed on separate benches to avoid the transfer of sound. Each consisted of a smaller experimental plastic tank (40 × 30 × 30 cm, 2 mm walls and 25 cm water depth) inside a larger plastic tub (70 × 50 × 60 cm, 3 mm walls and 25 cm water depth), with the speaker suspended in the larger tank to avoid contact with the sides. Two randomly allocated tanks received two different ambient playback tracks (details above) while the other two received different boat tracks. Fifty-eight fish were evenly split in an independent-measures design between the two sound treatments, with each weighted tube placed in the allocated exposure tank for the 30 min trial period. No significant differences were found in the weight (mean±s.e., ambient: 40.7±2.8 mg, boat: 42.9±0.8 mg, *t*-test: *t*=0.78, *P*=0.437) or size (mean±s.e., ambient: 11.7±0.1 mm, boat: 11.9±0.1, *t*-test: *t*=1.63, *P*=0.108) of fish allocated to the two treatments.

In the second experiment, a weighted plastic crate was placed on sand in 2 m water. *P. amboinensis* were transported to the test site as for the patch reef experiment (details above), before sealing in tubes that were then placed in the crate. In two blocks of trials (treatment order reversed on the second day of testing), fish were randomly allocated and exposed to either one of two boats driving at 10–200 m distance from the crate, or to ambient conditions. Thirty-six fish were evenly split in an independent-measures design between the two sound treatments, with each weighted tube placed in the crate for the 30-min trial period. No significant differences existed in the weight (mean±s.e., ambient: 40.5±1.4 mg, boat: 38.7±1.2 mg, *t*-test: *t*=0.94, *P*=0.351) or size (mean±s.e., ambient: 12.5±0.1 mm, boat: 12.5±0.1, *t*-test: *t*=0.47, *P*=0.640) of fish allocated to the two treatments.

### Impact of boat noise on anti-predator behaviour

We adapted the simulated ‘ambush predator' looming-stimulus experiment (details in ref. 18), which isolates the visual component of a predatory strike, for use in open-water conditions. The stimulus consisted of a 60-cm length of 22 mm PVC pipe with a black end cap that emerged from a larger pipe secured to a concrete block. The stimulus was remotely released and powered by a speargun rubber so that it travelled at high speed for 350 ms towards a 250-ml plastic holding pot. The stimulus, which appeared as a black disk increasing in size as it moved towards the fish, was prevented from hitting the holding pot by a lanyard.

*P. amboinensis* were collected, housed and transferred to the test site as described above. Individual fish were transferred by scoop into holding pots with fresh aerated seawater 15 min before the experiment to minimize stress. During this time, all fish returned to normal swimming behaviour and ventilation rates. For each trial, a pot with a fish was attached in position on a second concrete block for 1 min to acclimatise to the experimental arena on the seabed before release of the stimulus. An observer snorkelled at the surface (to avoid the noise of SCUBA) and was hidden from the fish behind a large plastic tub, on which an underwater video camera (HDR-XR520VE, 25 f.p.s., Sony Corporation) was positioned to film the experiment. After 1 min acclimatization, the stimulus was released.

In two blocks of trials (treatment order reversed on the second day of testing), fish were randomly allocated to be exposed to one of two different boats driving continually at 10–200 m distance from the experiment or to ambient conditions. Limited visibility (<10 m) meant that, when present, the boat could not be seen by the fish at the test site. Thirty fish were evenly split between the two treatments and blocks in an independent-measures design. The videos were analysed without sound (thus ‘blind' to the acoustic treatment), to determine whether *P. amboinensis* startled (a rapid shift in position or obvious directional change in swimming trajectory between consecutive frames^20^) in response to the looming stimulus. When the fish did startle, the time taken to startle (from initiation of looming-stimulus release) and the distance between the stimulus and the fish at the point of startle were also calculated.

### Impact of boat noise on strike–success rate of predators

We tested the effect of boat-noise playback on the interaction between settlement-stage *P. amboinensis* and the predator *P. fuscus*. Like many dottybacks, *P. fuscus* is resident in a small territory on coral heads, and hunts for newly settled damselfishes that typically shelter in the branches of a single coral head because of the risk of further relocation. Adult *P. fuscus* were collected from reefs >2.5 km to the east of our study site by divers using a dilute clove oil solution and hand nets, and kept separately in a flow-through aquarium system (subsurface inflow of water, no bubblers) for a minimum of 3 days before use in the experiment (as per ref. 24). Each day, *P. fuscus* were fed two live *P. amboinensis* that had been collected the previous night using light traps. The day before testing, *P. fuscus* were not fed; predators naturally feed episodically in the wild, and not feeding for 24 h ensured consistency between trials. *P. fuscus* were transferred by scoop (to minimize stress from handling) into six 40 × 30 × 30 cm plastic tanks (2 mm walls, 25 cm water depth; subsurface inflow of water, no bubbler) inside larger plastic tubs (70 × 50 × 60 cm, 3 mm walls and 25 cm water depth) on separate benches to avoid sound transfer. Each tank contained a live *Pocillopora damicornis* coral head (∼15 × 15 × 15 cm) collected locally. *P. fuscus* were given 24 h to acclimatize to their experimental habitat.

Settlement-stage *P. amboinensis* were collected using light traps and kept in holding tanks as above. Since they were collected before settlement and contact with reef habitat these fish were naïve to *P. fuscus*. Before testing, 10 *P. amboinensis* were transferred by scoop into a 1 l jug and allowed to recover for 15 min, during which time they resumed normal swimming behaviour and ventilation rates. An experimental arena was constructed from a smaller experimental plastic tank (40 × 30 × 30 cm, 2 mm walls, 25 cm water depth and acoustically transparent) inside a larger plastic tub (70 × 50 × 60 cm, 3 mm walls and 25 cm water depth). A speaker (details above) playing either an ambient or a boat track was suspended in the larger tank to avoid contact with the sides. The allocation of ambient or boat playback treatments was alternated between the experimental benches each day, and the order of the trials was counterbalanced to avoid any effects of time of day on behaviour.

At the start of each trial, 10 *P. amboinensis* were released into a section of the experimental tank separated from the *P. fuscus* by a transparent Perspex screen. After 30 s, the screen was lifted and the activities of predator and prey observed from behind a hide for 15 min. Every ‘strike' (targeted lunge) at a prey fish and ‘capture' (prey fish caught and eaten) were recorded. After 15 min, the *P. fuscus* was removed and placed in a polythene bag for measurement of size. No significant difference was found in the length (mean±s.e., ambient: 75.9±1.1 mm, boat: 76.0±1.2, *t*-test: *t*=1.23, *P*=0.221) of *P. fuscus* allocated to the two treatments. Thirty-six trials (six blocks of three trials per treatment) were conducted, with *P. fuscus* and *P. amboinensis* randomly allocated between treatments; predator and prey fish were only ever used once in the experiment.

### Impact of boat noise on mortality due to predation

Data on survival of prey during interactions with predators were collected from two experiments. The first was the tank-based experiment described above, in which the number of *P. amboinensis* remaining at the end of the 15 min trial was counted in addition to data on strike success of the predator. The second modified the tank experiment to test the impact of boat noise in the field. Here, trials were conducted in upturned 30 l plastic aquaria, each containing a live *Pocillopora damicornis* coral head (∼15 × 15 × 15 cm), placed on sheets of Perspex on the sand in 2–3 m of water; separate aquaria were at least 10 m apart.

Adult *P. fuscus* used in this experiment were collected and housed as described above. On the day of the experiment, *P. fuscus* that had not been fed for 24 h were carried to a shaded location on the shore in individual holding pots held in 60 l bins. One *P. fuscus* was placed into each arena and given 15 min to acclimatize to its surroundings; during this time, either one of two different boats was driven continually at 10–200 m distance from the arena (boat treatment) or the fish was exposed to ambient conditions. Five settlement-stage *P. amboinensis* (collected and housed as above) were transferred by scoop into a 500-ml plastic pot and given 15 min to recover from capture. They were then taken by snorkellers to the experimental arena and released. The snorkellers left the arenas immediately after release. After a treatment time of 15 min, the number of *P. amboinensis* remaining in the aquaria was counted by snorkellers. All fish were then removed and the size of each *P. fuscus* measured. The order of trials was alternated between the two days of experiments to avoid any effects of time of day on experimental outcomes. No significant differences were found in size (mean±s.e., ambient: 75.2±1.1 mm, boat: 75.1±1.5, *t*-test: *t*=0.07, *P*=0.942) of *P. fuscus* allocated to the two treatments. Forty-four trials (two blocks of 11 trials per treatment; order of treatments alternated between blocks) were conducted, with *P. fuscus* and *P. amboinensis* randomly allocated between treatments; predator and prey fish were only ever used once.

## Additional information

**How to cite this article:** Simpson, S. D. *et al.* Anthropogenic noise increases fish mortality by predation. *Nat. Commun.* 7:10544 doi: 10.1038/ncomms10544 (2016).
